# Iron Overload and Chelation Therapy in Non-Transfusion Dependent Thalassemia

**DOI:** 10.3390/ijms18122778

**Published:** 2017-12-20

**Authors:** Rayan Bou-Fakhredin, Abdul-Hamid Bazarbachi, Bachar Chaya, Joseph Sleiman, Maria Domenica Cappellini, Ali T. Taher

**Affiliations:** 1 Department of Internal Medicine, American University of Beirut Medical Center, Beirut 11-0236, Lebanon; rib05@mail.aub.edu (R.B.-F.); joseph.sleiman.22@gmail.com (J.S.); 2 Faculty of Medicine, American University of Beirut Medical Center, Beirut 11-0236, Lebanon; aab42@mail.aub.edu (A.-H.B.); bfc01@mail.aub.edu (B.C.); 3 Department of Medicine, Ca’Granda Foundation IRCCS, University of Milan, 20122 Milan, Italy; maria.cappellini@unimi.it; 4 Department of Clinical Science and Community, University of Milan, 20122 Milan, Italy

**Keywords:** non-transfusion dependent thalassemia, iron overload, iron chelation therapy, serum ferritin, liver iron concentration

## Abstract

Iron overload (IOL) due to increased intestinal iron absorption constitutes a major clinical problem in patients with non-transfusion-dependent thalassemia (NTDT), which is a cumulative process with advancing age. Current models for iron metabolism in patients with NTDT suggest that suppression of serum hepcidin leads to an increase in iron absorption and subsequent release of iron from the reticuloendothelial system, leading to depletion of macrophage iron, relatively low levels of serum ferritin, and liver iron loading. The consequences of IOL in patients with NTDT are multiple and multifactorial. Accurate and reliable methods of diagnosis and monitoring of body iron levels are essential, and the method of choice for measuring iron accumulation will depend on the patient’s needs and on the available facilities. Iron chelation therapy (ICT) remains the backbone of NTDT management and is one of the most effective and practical ways of decreasing morbidity and mortality. The aim of this review is to describe the mechanism of IOL in NTDT, and the clinical complications that can develop as a result, in addition to the current and future therapeutic options available for the management of IOL in NTDT.

## 1. Introduction

Thalassemia is an inherited autosomal recessive hemoglobin disorder. At the molecular level, these disorders stem from a defect either on chromosome 11 (encoding the β chain globin) or on chromosome 16 (encoding the α chain globin) leading to myriad phenotypes (β-thalassemia and/or α-thalassemia, respectively) [1]. Over the years, study of the thalassemia syndromes has served as a paradigm for gaining insights into the cellular and molecular biology, as well as the pathophysiology, of inherited genetic disorders. Most patients with thalassaemia are born in resource-poor countries, but modern migration patterns have altered the epidemiology of this disease, and patients with thalassaemia are now found in areas such as Europe and North America.

The disease hallmarks of thalassemia include an imbalance in the α/β-globin chain ratio, leading to ineffective erythropoiesis, chronic haemolytic anaemia, and iron overload. These pathophysiological mechanisms lead to an array of clinical manifestations involving numerous organ systems. The degree of transfusion dependence is one of the elements considered in a recent classification of thalassemic disorders into transfusion-dependent thalassemia (TDT) and non-transfusion dependent thalassemia (NTDT).

The NTDT class comprises several thalassemia syndromes, including β-thalassemia intermedia (β-TI), hemoglobin H disease, and mild-to-moderate forms of hemoglobin E/β-thalassemia. Though they are transfusion independent, patients with NTDT can still require transfusion therapy sporadically, or even regularly, for the prevention or management of certain disease manifestations. Iron overload (IOL) is one of the main culprits of disease-related morbidity in NTDT. It is one of the most prominent clinical manifestations in NTDT. Iron overload in NTDT patients is a cumulative process with advancing age, and concerns over secondary morbidities start beyond the ages of 10–15 years [1,2,3,4,5]. Despite receiving no or only occasional blood transfusions, patients with NTDT have increased intestinal iron absorption, and can accumulate iron to levels comparable with TDT patients. This iron accumulation occurs more slowly in NTDT patients compared to TDT patients, and contributes in some way to the severity of some complications [3]. The understanding of the underlying pathophysiological mechanisms of IOL in NTDT and its associated clinical morbidity has increased in recent years. This new knowledge, in addition to the increasing awareness of the limitations of current ICT strategies, is driving research into seeking novel therapeutic options for these patients, with amelioration of ineffective erythropoiesis and iron dysregulation as the key targets for patients with NTDT. The aim of this review is to describe the mechanism of IOL in NTDT, and the clinical complications that can develop as a result, and the current and future therapeutic options for the management of IOL in NTDT.

## 2. Mechanism of Iron Overload (IOL) in Non-Transfusion-Dependent Thalassemia (NTDT)

**Figure 1:**
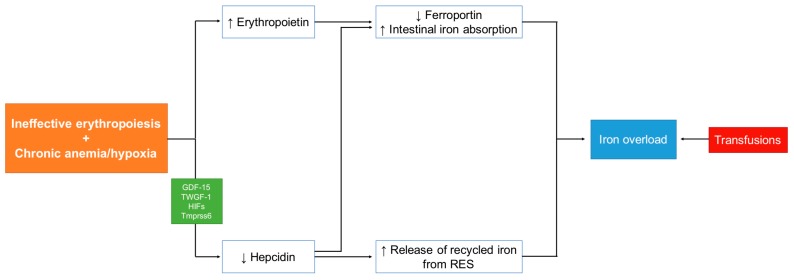
Iron overload mechanism in non-transfusion-dependent thalassemia. GDF-15: growth differentiation factor-15; TWGF-1: twisted gastrulation factor-1; HIFs: hypoxia inducible transcription factors; TMPRSS6: transmembrane protease, serine 6. (↑: increase; ↓: decrease).

The mechanism of increased intestinal iron absorption in NTDT patients is triggered by a cascade that is initiated by ineffective erythropoiesis [6,7]. The conditions of anemia and hypoxia that result from ineffective erythropoiesis influence the expression of the serum protein hepcidin, which is the main regulator of intestinal iron absorption in the body [8,9]. Hepcidin negatively regulates iron absorption because it downregulates the expression ferroportin, a transmembrane protein responsible for iron absorption from the gastrointestinal tract and for exporting intracellular iron into circulation [10]. Hepcidin levels decline when iron sequestration for erythropoiesis increases [8], and this, in turn, results in upregulated ferroportin. High levels of ferroportin will then cause an increased release of iron from the reticuloendothelial system, leading to depletion of macrophage iron [11,12]. The downregulation of hepcidin in NTDT can also be mediated by growth differentiation factor-15 (GDF-15) [11,13,14] and twisted gastrulation factor [15]. New studies have highlighted the role of GDF-15 in creating the state of iron overload in NTDT. GDF-15 is a member of the transforming growth factor-β (TGF-β) family and is normally upregulated during ineffective erythropoiesis, causing the downregulation of hepcidin. Since NTDT is a disease of ineffective erythropoiesis, high blood levels of GDF-15 have been noted is these patients [16]. Figure 1 portrays the mechanism of IOL in NTDT.

## 3. Diagnosis and Quantification of IOL

Several modalities are currently available for the diagnosis and monitoring of IOL in NTDT, each carrying their own advantages and disadvantages. Serum ferritin (SF) assessment is widely available and is heavily relied upon in resource-poor countries [17]. Moreover, the assessment of serial SF concentrations can also be a good indicator of iron chelator effectiveness [18]. SF levels, however, are lower in NTDT than TDT for the same degree of hepatic iron concentration, and thus they underestimate the iron burden [19]. In NTDT patients, SF values of >800 μg/L and <300 μg/L are associated with an increased and absence of risk for morbidity, respectively [1,4]. Non-invasive iron monitoring using R2 or T2* techniques by MRI has replaced liver biopsy as the gold standard for the quantification of liver iron concentration (LIC) given its safety and reliability. In NTDT patients, LIC values >5 mg/g are associated with increased morbidity, supporting the recommendation of ICT initiation in patients with LIC levels >5 mg/g [20]. As per the available guidelines, assessment of LIC should be performed every 6, 12, or 24 months [1]. This will depend on the severity of the iron overload. Moreover, results from the ORIENT study revealed that patients with SF ≥800 μg/L have a higher incidence of morbidities over 11 years [4]. Based on a ROC analysis, a SF level of ≥800 μg/L had the highest accuracy for predicting LIC ≥5 mg Fe/g dry weight (dw) [21].

## 4. Morbidities Secondary to IOL

NTDT is associated with a high morbidity profile that can start manifesting as early as 10 years of age [22]. Increased iron burden and accumulation underlies some of the complications seen in NTDT, and contributes to their severity. This iron burden promotes oxidative damage in a variety of organs, inducing a variety of endocrine and end organ dysfunction [23]. In the liver, chronic iron deposition is detrimental to hepatocytes and can result in fibrosis, cirrhosis and eventually hepatocellular carcinoma [24]. Furthermore, endocrine glands have a high sensitivity for iron toxicity, and thus, iron deposition in these glands (thyroid, parathyroid, testicles, pancreas etc.) may result in hypothyroidism, hypoparathyroidism, hypogonadism, and diabetes mellitus type 1, respectively [2,3,22,25,26]. Kidneys are also affected by iron overload, and this can manifest as both proteinuria and glomerular hyperfiltration as a consequence of tubulointerstitial and glomerular damage, and patients can be at risk of end-stage renal disease [27]. Iron overload, in addition to nutritional imbalance and increased erythron (due to ineffective erythropoiesis), can also lead to osteoporosis, osteopenia, and other low bone mineral density states in patients with NTDT [28,29]. There is also evidence for an association between IOL and vascular disease in NTDT patients. Increased LIC was associated with a higher prevalence of thrombosis and pulmonary hypertension in a cross-sectional analysis of β-TI patients, and in splenectomized adults, there is a relationship between IOL and cerebrovascular disease [30,31]. However, we now know that in NTDT patients, hypercoagulability is the main contributor in the development of vascular complications. Finally, IOL cardiomyopathy is a rare complication of NTDT and is more routinely associated with TDT [32]. The phenotypic diversity of NTDT results from its underlying genetic diversity, which can be explained by primary, secondary or tertiary modifiers [33,34]. Because of this, the amount of iron accumulation could vary across different genotypes, thus not only affecting the frequency, but also the severity of IOL-related morbidities.

## 5. Iron Chelation Therapy

Despite all the recent advances in the understanding of pathophysiology and the development of new novel therapies, iron chelation therapy (ICT) remains the backbone of NTDT management, and is one of the most effective and practical ways to decrease morbidity and mortality [23]. Today, the primary goal of ICT has shifted from treating or rescuing IOL to maintaining safe levels of body iron at all times [18]. To achieve this, iron intake must be balanced with iron excretion by chelators to prevent iron accumulation and end-organ complications and lead to normal survival and improved quality of life. Therefore, appropriate tailoring of ICT with chelator choices and dose adjustments must be implemented in a timely manner. Moreover, the clinical decision to initiate, adjust and stop ICT is based on SF, MRI-LIC and cardiac T2*.

Currently three iron chelators are widely available and used: deferoxamine (DFO) in subcutaneous or intravenous injection; oral deferiprone (DFP) in tablet or solution form; and oral deferasirox (DFX), in dispersible tablet (DT) and—more recently—film-coated tablet (FCT) forms [1,18,35]. While all three of these drugs have proven their effectiveness as iron chelators in TDT patients, DFX remains the only drug that has received Food and Drug Administration (FDA) and European Medicines Agency (EMA) approval for use in NTDT patients, mostly based on results extracted and published from the THALASSA trial [2,36]. In this multinational, prospective, randomized, double-blinded phase II trial conducted on 148 patients comparing DFX (97 patients) to placebo (51 patients) [36], 1-year treatment of NTDT patients older than 10 years was found to decrease LIC by a mean of 2.33 ± 0.70 and 4.18 ± 0.69 mg Fe/g dry weight at a daily dose of 5 and 10 mg/kg, respectively, compared to placebo [36]. Sub-analyses further proved that irrespective of baseline LIC, SF, underlying NTDT form, splenectomy status or demographics such as age, gender and race, both DFX 5 and 10 mg/kg/day starting doses led to consistent reductions in LIC across all patients [36]. The analyses also showed that greater reductions in LIC were achieved in patients dose-escalated at 6 months from DFX 10 mg/kg/day starting dose to 20 mg/kg/day [36]. There were no adverse effects noted in patients who received DFX compared to placebo, and the only major side effects reported—mild to moderate in severity, and self-resolving without drug discontinuation—were nausea/gastrointestinal discomfort and headache [36]. A 1-year extension phase was then carried out to allow for the assessment of up to 2 years of DFX treatment. Patients continued to respond, with a decrease in LIC and SF over 2 years. Data extracted from the THETIS study [37] further showed that a starting dose of 10 mg/kg/day of DFX is effective in reducing iron overload in NTDT, and that dose escalation up to 30 mg/kg/day should be considered starting at week 4 based on LIC response [37]. The THETIS analyses also strengthened previous evidence concerning the safety profile of DFX while also providing insight into its possible association with increased risk of pancreatitis and ocular toxicity [37]. DFP has not been extensively studied in NTDT. Single-arm, open-label studies with small sample sizes and a more recent randomized controlled trial showed significant decreases in SF and LIC with DFP therapy [38]. DFO has not been systematically studied in NTDT, although studies with small sample sizes and short durations have shown an increase in urinary excretion of iron and a decrease in SF.

**Figure 2:**
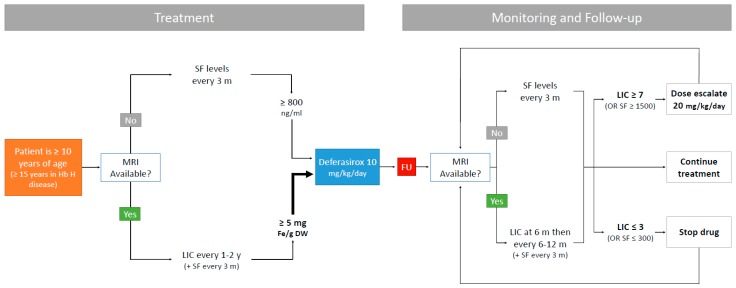
Iron overload screening, diagnosis and treatment algorithms in non-transfusion-dependent thalassemia [1,37]. SF: serum ferritin; LIC: liver iron concentration in mg Fe/g dry weight; FU: follow-up.

ICT is indicated for virtually all TDT patients receiving regular transfusions, but with NTDT, specific indications have been established for its initiation, dose escalation and termination [1]. The Thalassemia International Federation (TIF) recommends that DFX chelation with initial starting dose of 10 mg/kg/day should be started in patients ≥10 years of age (15 years of age in hemoglobin H disease) if their LIC ≥5 mg Fe/g dry weight, or if their SF concentration was found to be ≥800 μg/L when LIC is not available due to lack of the necessary MRI technology [1]. As for monitoring of iron levels, LIC should be repeated 6 months after therapy initiation, with follow up every 6–12 months, in addition to SF levels being measured every 3 months [1]. If at 6 months LIC is still >7 mg Fe/g dry weight (or SF >1500 μg/L only if LIC is unavailable) with less than 15% reduction in baseline values, dose escalation should be considered up to 20 mg/kg/day [1]. DFX therapy can be safely discontinued when patients reach an LIC value of 3 mg Fe/g dry weight (or SF level of 300 μg/L only if LIC is unavailable) [1]. In NTDT, it is recommended to intensify ICT if the LIC after 6 months of treatment >7 mg/g dw. liver or SF >1500–2000 ng/mL and <15% decrease from baseline. Indications to stop ICT in NTDT include a SF < 300 ng/mL and/or LIC < 3 mg/g dry wt. liver. Figure 2 portrays a screening, diagnosis, and treatment algorithm for IOL in NTDT.

## 6. Recent Advances in ICT

Compliance with ICT is associated with effective control of iron overload and improved patient survival [39,40,41]. While iron chelation has proven itself as both a safe and effective for NTDT patients, a major issue of compliance stands in the way of their successful management and ensuring adherence in these patients should be of upmost priority [42]. The use of DFX dispersible tablets (DTs) automatically implies several inconveniences to the patient including palatability, the need to take the drug in a fasting state (i.e., not being able to take it with food), and drug-related side effects, notably gastrointestinal (GI) tolerability [42]. These factors have created a barrier to optimal adherence to DFX DTs, which has prompted the development of a new film-coated tablet (FCT) formulation [42]. This formulation can be swallowed once-daily, both whole or crushed, and regardless of whether the patient is fasting or not [42]. The open-label, phase II ECLIPSE study also evaluated patient-reported outcomes (PRO) in TDT or lower-risk myelodysplastic syndromes patients randomized to receive DFX DT or FCT over a 24-week [42]. FCT recipients were uniformly more satisfied, and had fewer concerns with the drug, and even reported better adherence and compliance, all while preserving the great safety profile that DT formulations of DFX are well known for [42]. This new formulation provides a promising alternative that would increase patient satisfaction and adherence, ensure better treatment outcomes, and overall contribute to a better quality of life [42].

## 7. Novel Therapies

Newly emerging therapies targeting iron dysregulation include minihepcidins, transmembrane protease serine 6 (TMPRSS6).

### 7.1. Minihepcidins

Minihepcidins, or long-acting hepcidin analogs, have been shown to restrict iron absorption, and their utilization in the setting of IOL has shown beneficial effects on ineffective erythropoiesis [43,44,45]. They are known to increase the levels of endogenous hepcidin, thereby decreasing iron absorption from the GI tract, increasing the redistribution of iron to macrophages, and limiting end-organ toxicity [46]. Studies on mice have also shown that minihepcidin therapy not only increases Hb concentrations, but also decreases reticulocyte counts and reduces spleen size [46,47].

### 7.2. TMPRSS6

Encouraging preclinical data on transmembrane protease serine 6 (TMPRSS6) inhibitors have also been recently reported, as an approach to stimulate endogenous hepcidin production [48,49,50]. TMPRSS6 is a transmembrane serine protease that reduces production of hepcidin. Thus, endogenous hepcidin production can be stimulated by reducing expression of TMPRSS6. Data from mouse models suggest that deletion of the TMPRSS6 gene improves anaemia and reduces ineffective erythropoiesis, splenomegaly, and iron loading [48]. Use of antisense oligonucleotides or small interfering RNAs that target TMPRSS6 has been shown to improve anaemia and iron overload in mice and other preclinical models of β-thalassaemia [49,50,51]. Genetic ablation of TMPRSS6 also improved ineffective erythropoiesis and decreased splenomegaly in β-TI, without a concomitant decrease in erythropoietin production [48]. Normalization of RBC survival is a significant component of the effects of TMPRSS6 inhibition on both hemoglobin and spleen size. The above-mentioned data on TMPRSS6 provide proof of principle that pharmacologic manipulation of hepcidin may be an effective treatment for human diseases of iron dysregulation.

## 8. Conclusions

In conclusion, IOL due to increased intestinal iron absorption represents an important clinical problem in NTDT patients, particularly as they advance in age. Adequate assessment and monitoring of NTDT patients, in addition to tailored ICT, is crucial for preventing the complications known to be associated with this increased iron burden. New treatment modalities are currently being investigated to broaden options available for NTDT management, with ultimate goals of prolonging longevity, promoting greater compliance and better adherence and improving quality of life. Since NTDT patients present with multiple pathophysiologies, tailoring treatment will always remain essential. Therefore, future studies should aim at creating a validated tool that can be used to assess IOL severity and tailor therapy, allowing for standardization of assessments that would lead to timely interventions and prevention of IOL-related complications.

## Abbreviations

IOL	Iron overload
NTDT	Non-transfusion dependent thalassemia
ICT	Iron chelation therapy
TDT	Transfusion-dependent thalassemia
β-TI	β-thalassemia intermedia
GDF-15	Growth differentiation factor-15
TGF-β	Transforming growth factor-β
SF	Serum ferritin
LIC	Liver iron concentration
dw	dry weight
DFO	Deferoxamine
DFP	Deferiprone
DFX	Deferasirox
DT	Dispersible tablet
FCT	Film-coated tablet
EMA	European Medicines Agency
TIF	Thalassemia International Federation
GI	Gastrointestinal
PRO	Patient-reported outcomes
TMPRSS6	Transmembrane protein serine 6
